# Colloid Cyst of Appendix

**Published:** 1915-10

**Authors:** 


**Colloid Cyst of Appendix.** A. F. Tyler of Omaha, Interstate Med. Jour., Sept., states that while this condition is rarely reported by surgeons, it was found in 4% of necropsies. Batas of New Orleans has reported a cyst weighing 52.7 ounces and measuring 18¾x17½x4 inches. This was a psuedomucinous cyst with the appendix attached to one side. (Apparently this would come under the classification of mesenteric cyst.—Ed.) Tyler's case occurred in a married woman, with symptoms of appendicitis. Blood was found in the stools on a meat-free diet. Roentgen examination showed the cyst to be retrocaecal. On operation, it proved to be a cystic dilatation of the appendix, the contents resembling white of egg. The cyst was 4x1 inch in its thickest part and fusiform.

